# Characteristics of Nationwide Urinary Tract Infection (UTI) Visits by Age and Type II Diabetes Status in Women

**DOI:** 10.7759/cureus.46000

**Published:** 2023-09-26

**Authors:** Sara B Papp, Alana L Christie, Philippe E Zimmern

**Affiliations:** 1 Department of Urology, University of Texas (UT) Southwestern Medical Center, Dallas, USA

**Keywords:** nationwide, diabetes mellitus type 2, urinary tract infection, aging, women

## Abstract

Background

Through a national database search of office visits, we studied the contribution of two known risk factors for urinary tract infections (UTIs) in women: age and type 2 diabetes mellitus (T2DM).

Methodology

The National Ambulatory Medical Care Survey (NAMCS) database was queried for visits including a UTI diagnosis and a urine culture order. Data were included for all visits involving adult women for available years, 2014-2016 and 2018. Data on demographics, reason for visit, T2DM status, UTI workup, and UTI treatment were collected. Patients with Alzheimer’s disease or chronic kidney disease were excluded. Descriptive statistics were displayed as weighted means with standard errors for continuous variables. The effect of age was compared based on a 65-year-old cutoff.

Results

One hundred sixty-seven surveyed visits were analyzed for the years 2014-2016 and 2018, representing an estimated 7.4 million visits nationwide. Women ≥65 years were more likely to be white, non-Hispanic/non-Latino, from the Midwest or West, from metropolitan areas, and on Medicare/Medicaid than their younger counterparts. T2DM and urinalysis rates did not significantly vary between the two age groups (7.7% vs. 14.6%, *P* = 0.3; 78% vs. 76%, *P* = 0.9, respectively). For urinalysis rates between patients with and without T2DM, there was no significant difference in the <65-year-old group (80% vs. 78%, *P* = 0.9) or the ≥65-year-old group (93% vs. 73%, *P* = 0.12). Antibiotic prescription rates were also similar for T2DM and non-T2DM patients (67% vs. 75%, *P* = 0.7).

Conclusions

Through a national database analysis, we reported the demographic and visit differences aged <65 years and ≥65 years who sought care for UTIs in the United States over a four-year period. T2DM rates and urinalysis did not vary between age groups, and urinalysis rates and antibiotic prescription rates did not vary between T2DM and non-T2DM groups in an age-dependent matter. More research is needed to understand the demographic makeup and risk factors of UTI patients across the nation.

## Introduction

Urinary tract infections (UTIs) are common infections. Over 10% of women are diagnosed annually in the United States with a UTI, and over 60% of women have at least one UTI in their lifetime [1,2]. Two factors that contribute to higher rates of UTIs are older age and type 2 diabetes mellitus (T2DM) [3,4]. Two large studies have shown that individuals with diabetes are more than 1.5 times more likely to be affected by UTIs than non-diabetic individuals and are more than twice as likely to have a drug-resistant uropathogen [5-9]. In addition, women aged 65 years or older are at increased risk for UTIs as summarized in a 2020 review [10]. Although these factors have been shown to contribute to higher UTI rates, the exact role they might play in the workup and treatment of UTIs in the United States is not well established.

With the high prevalence of antibiotic-resistant uropathogenic bacteria, there is an urgent need to optimize UTI diagnosis and treatment, especially in those who suffer from recurrent UTIs (rUTIs) [11,12]. As a result, we aimed to identify patterns in UTI diagnosis in a national database over four years. Through an age group analysis (<65 years versus ≥65 years), we studied how older age and T2DM status, prevalent conditions in the United States at the moment, correlate with urinalysis order rates and antibiotic prescription rates in UTI visits. In addition, we studied the demographic differences and visit characteristics of UTIs in the United States between women aged <65 and ≥65 years.

## Materials and methods

Data source

The National Ambulatory Medical Care Survey (NAMCS), an open-access database from the National Center of Health Statistics at the Center for Disease Control and Prevention, was used for this study. Visits with a UTI diagnosis and urine culture order (defined as any visit with an International Classification of Diseases (ICD) code for UTI and urine culture ordered at the time of visit) were analyzed for available years, 2014-2016 and 2018 (2017 data not yet released by NAMCS due to the COVID-19 pandemic). Details on the survey methodology can be found on the NAMCS website [13]. Data included various visit characteristics to examine how UTIs are worked up, diagnosed, and treated nationally. Age and T2DM status were additionally examined to see if they correlated with other study variables. Data collected were weighted to produce national estimates that account for potential sources of error. 

Study population

Our study included office visits from 2014 to 2016 and 2018 of female patients aged 18 years or older who had ICD, Ninth Revision (ICD-9) or ICD, Tenth Revision (ICD-10) codes relating to UTI (ICD-9: 599.0; ICD-10: N39.0) and had a urine culture ordered or provided at the visit. Only visits with urine cultures were included to better select for symptomatic UTIs, as urine cultures are typically ordered by providers for symptomatic UTIs only to guide antibiotic therapy. Patients who were male, younger than 18 years old, or had Alzheimer’s disease or chronic kidney disease were excluded. Up to five diagnosis codes were assigned based on the chief complaints at the time of the visit, so patients were identified as having a UTI-related problem if any of the five codes were for UTI. The major reason for the visit was categorized as a chronic problem (routine visit or flare-up, onset ≥3 months ago), new/acute problem (onset <3 months ago), pre/post-surgery visit (care required for surgery), preventative care (general/routine exams), or unknown reason. Data were obtained on patient age, race, ethnicity, region, payer type, obesity (body mass index [BMI] greater than 30 kg/m^2^), and T2DM status, as well as whether the patient was established, referred, seen by a physician, and/or from a metropolitan area. Additionally, workup details (urinalysis) and antibiotics prescribed were included, but urinalysis and urine culture results were not provided. Therefore, we understood that the surveyed physician in the NAMCS database met with a patient with UTI symptoms based on diagnostic codes (severity unknown), ordered a urinalysis, and opted to send the urine for culture. We assume that the physician was sufficiently concerned during the visit about the patient’s symptoms and urinalysis findings to order a urine culture to guide subsequent therapy. Since the urine culture was ordered before the antibiotic was prescribed, we assumed that the culture returned positive and guided the physician to the appropriate antibiotic choice.

Statistical analysis

Descriptive statistics were displayed as weighted means with standard errors for continuous variables, such as patient demographics and clinical characteristics of patient visits. Weighted frequencies of visits concerning UTI diagnosis were calculated by year, where a particular record is weighted to represent thousands of visits. This sampling process is provided by the NAMCS and is intended to provide a random sample of the types of visits that occur across the United States, with stratification including Census region, physician specialty, and metropolitan versus rural area. This weighting was calculated based on the likelihood of a particular visit to be sampled based on the annual U.S. Census Bureau postcensal population estimates. A full description of the sampling process is readily available at no cost on the NAMCS website [14]. The distribution of the estimated number of visits by age group was visualized as a scatter plot with error bars equivalent to the standard deviation. To evaluate the effect of age, a separate analysis was conducted comparing those above 65 years old (the usual cutoff for the geriatric population) and the rest of the group. All analyses were performed in SAS 9.4 (SAS Institute, Cary, NC, USA).

## Results

Our query found 167 surveyed visits for women at least 18 years old with a UTI diagnosis and a urine culture, representing an estimated 7.4 million visits based on the likelihood of each visit to have been surveyed (Table 1).

**Table 1:** Visit distribution by age group.

Age group	<65 years	≥65 years
Surveyed visits	85	82
Estimated total visits	4,038,207	3,377,626

**Figure 1:**
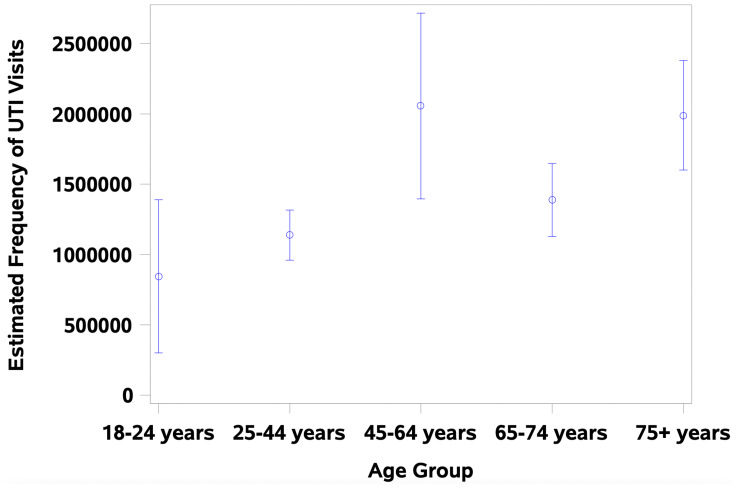
Age distribution of UTI diagnoses, 2014-2016 and 2018. UTI, urinary tract infection

Of the 167 surveyed visits, 85 (50.90%) were in the <65 years age group and 82 (49.10%) were in the ≥65 years age group. The estimated frequency of visits by age group is shown in Figure 1. The estimated frequency of visits with UTI diagnoses was relatively stable by year.

Results by category are summarized in Table 2. A higher rate of white patients was observed in those ≥65 years old (*P* = 0.0037). Patient ethnicity results also varied by age group with more women <65 years being Hispanic or Latino and more women ≥65 years being non-Hispanic/non-Latino (*P* = 0.015). Similarly, region (*P* = 0.040) and payer type (*P* < 0.0001) distributions varied significantly between age groups. Although not significantly associated, our analysis showed that 48% of patients <65 years cited a new/acute problem as the primary reason for their visit, compared to only 29% of patients aged 65 or older. In contrast, chronic problems were more commonly reported among those aged 65 or older, with 56% listing them as the major reason for their visit, compared to only 46% in those under the age of 65 (*P* = 0.14).

**Table 2:** Visit characteristics with UTI diagnosis by age group in adult women, 2014-2016 and 2018. *Region data missing for 2018. **Payer type analyzed without the *Self-pay* group, given the zero cells. Stderr, standard error; UTI, urinary tract infection; NE, not estimated

Category	<65 years (Percentage ± Stderr)	≥65 years (Percentage ± Stderr)	*P*
Race			
Black	5.2 ± 1.9	2.3 ± 0.3	0.0037
Other	1.2 ± 0.7	1.9 ± 1.2	
White	57.5 ± 8.6	79.7 ± 5.3	
Unknown	36.1 ± 9.2	16.1 ± 5.1	
Ethnicity			
Hispanic or Latino	23.2 ± 7.0	3.9 ± 2.2	0.015
Non-Hispanic/Non-Latino	67.0 ± 7.8	83.4 ± 4.9	
Unknown	9.8 ± 4.4	12.7 ± 4.7	
Major reason for the visit			
Chronic problem	45.9 ± 12.3	55.5 ± 6.8	0.14
New/acute problem	48.3 ± 11.3	28.7 ± 5.5	
Pre-/post-surgery	0.7 ± 0.7	7.4 ± 5.6	
Preventive care	3.2 ± 0.6	3.7 ± 0.9	
Unknown	1.9 ± 1.6	4.7 ± 0.6	
Patient referred?			
No	29.6 ± 5.0	27.8 ± 3.8	0.6
Yes	20.6 ± 7.7	27.4 ± 5.9	
Unknown	49.8 ± 10.3	44.8 ± 6.3	
Region^*^			
Midwest	12.1 ± 4.2	26.6 ± 5.6	0.04
Northeast	26.1 ± 11.3	18.9 ± 8.5	
South	50.1 ± 10.1	34.5 ± 5.8	
West	11.8 ± 3.2	20.3 ± 4.4	
Metropolitan area	87.7 ± 4.5	99.2 ± 0.6	0.0014
Payer type^**^			
Unknown	23.5 ± 12.1	0.8 ± 0.8	<0.0001
Medicare/Medicaid	9.2 ± 3.5	89.7 ± 2.0	
Private insurance	65.6 ± 11.6	9.5 ± 1.8	
Self-pay	1.7 ± 1.1	NE	
Established patient	85.1 ± 3.5	89.8 ± 3.2	0.3
Seen by physician	99.6 ± 0.1	100.0 ± 0.0	-
Type 2 diabetes	7.7 ± 4.1	14.6 ± 2.2	0.3
Obese	3.3 ± 1.6	2.8 ± 1.2	0.8
Urinalysis	77.8 ± 10.9	76.1 ± 9.0	0.9
Antibiotic prescribed	77.7 ± 4.9	69.7 ± 8.0	0.4
Antibiotic			
None	22.3 ± 4.9	30.3 ± 8.0	0.5
Cephalosporin	0.9 ± 0.8	1.5 ± 0.7	
Nitrofurantoin	5.6 ± 1.9	2.6 ± 1.0	
Penicillin	5.8 ± 3.7	13.9 ± 2.1	
Quinolone	28.2 ± 5.1	28.4 ± 5.6	
Sulfonamide	14.2 ± 6.8	8.9 ± 4.0	
Other	0.6 ± 0.5	0.3 ± 0.0	
Multiple families	22.4 ± 10.2	14.2 ± 5.8	

T2DM rates did not significantly vary between the two age groups (7.7% vs. 14.6%, *P* = 0.3). Urinalysis rates did not significantly vary between patients <65 years and patients ≥65 years (78% vs. 76%, *P* = 0.9). The urinalysis order rates were similar for patients with and without T2DM in the <65 groups vs. 78%, *P* = 0.9). Similarly, for the ≥65 groups, we did not observe a difference in the rate of urinalysis orders in the patients with T2DM compared to those without (93% vs. 73%, *P* = 0.12). We also did not observe a difference in antibiotic prescription rates between T2DM patients and non-T2DM patients (67% vs. 75%, *P* = 0.7) or between age groups (77.7% vs. 69.7%, *P* = 0.4). Finally, demographic and visit data, including patient referral rates (*P* = 0.6), established patient rates (*P* = 0.3), and obesity rates (*P* = 0.5) were not different based on age group. 

## Discussion

This study investigated demographic variations among UTI patients across different age groups. It also analyzed UTI visit urinalysis rates and antibiotic prescription rates based on both T2DM status and age, utilizing data from the NAMCS database. This large and freely accessible national database offers a unique opportunity to review the management of UTIs by a geographically diverse group of surveyed physicians. We analyzed 167 surveyed visits and found significant differences between women aged <65 and ≥65 years in race, ethnicity, region, metropolitan area, and payer type. Although not statistically significant, we found that the younger age group had more new/acute UTI visits, while the older age group had more chronic UTI visits. T2DM rates for individuals with UTI diagnoses did not vary significantly between the <65 age group and the ≥65 age group. This finding suggests that T2DM is not correlated with UTIs in an age-dependent manner in our dataset. Neither age group in our study had drastically different T2DM rates compared to national T2DM rates, around 11.3%, as reported by the CDC [15]. We expected higher rates of T2DM in our populations because of the well-established notion that individuals with T2DM are at an increased risk for UTIs [4]. This finding could result from our limited sample size, especially for individuals with T2DM. In addition, excluding patients with various diseases (such as chronic kidney disease) that share comorbidities with T2DM may also have contributed to our low T2DM rates.

Our study found that urinalysis rates did not vary significantly by age in patients presenting with UTI symptoms. In addition, no significant differences were found when comparing urinalysis rates by T2DM status within each age group. This suggests that providers were not influenced by diabetes status to obtain a urinalysis at the time of the visit. We also did not observe a difference in antibiotic prescription rates between patients with and without T2DM. This is likely because guidelines require urine culture results before assigning antibiotic treatment in all populations. However, our finding is significant as it suggests that providers are not prematurely treating urinary tract infections based on the risk factors of older age and T2DM. These findings indicate that neither age nor T2DM status alone influences whether providers order urinalysis tests or prescribe antibiotics for adult female patients with symptomatic UTIs. Once again, our exclusions of patients with certain comorbidities may have influenced these results. 

We found significant differences in race, ethnicity, and region between women aged <65 years and ≥65 years with UTIs. A higher rate of white patients, non-Hispanic/non-Latino patients, and patients from the Midwest and West were observed in those aged ≥65 years. In contrast, obesity rates, patient referrals, and established patient status did not differ between age groups. These demographic data provide valuable insights into patterns of UTIs across the nation. Finally, payer type varied significantly between age groups, as a high percentage of women aged ≥65 years had Medicare/Medicaid. This finding is likely the result of the Medicare system covering individuals over 65 years of age.

Chronic problems were listed as the major reason for visits in 56% of patients aged ≥65 years and 46% in those aged <65 years. These rates are comparable to findings in the literature examining rUTI and diabetes. A study by Bonadio et al. in 2000 found that in diabetic individuals with a culture-confirmed UTI, 52.8% of cases were recurrent infections, while the recurrence rate was 42.9% in the non-diabetic group, although the difference did not reach statistical significance [16]. Another study from 2019 by Vinod et al. found that 14.4% of UTI cases were recurrent in diabetic individuals with urine culture-confirmed UTIs, while this rate was 10.5% in the non-diabetic group [17]. The differences between these rates and our findings are likely due to variations in study populations as both Bonadio et al. and Vinod et al. included both type 1 and type 2 diabetics, did not exclude men, and used patient populations of varying age ranges. In addition, neither study examined UTI recurrence rates independent of diabetes or by age group, while our study looked at T2DM rates only by age group. A study that examined the overall UTI recurrence rate in premenopausal women with an established UTI diagnosis is the 2015 study by Nseir et al. [18]. While the aim of this study was similar to ours, it included younger patients (ages 20-55) and defined recurrent UTIs differently than the NAMCS defined as a chronic issue. NAMCS provided category of *chronic* problem is determined purely by disease timeline (>3 months) and is not the same as the widely accepted criteria for rUTIs of two symptomatic UTI episodes within six months or three episodes within a year [19].

This study’s strengths include the use of a national database that provided a heterogeneous sample of UTI diagnoses from providers nationwide. By excluding visits with ICD codes for UTI but no urine culture ordered at the same visit, we obtained a refined cohort that included women who were more likely to have UTI symptoms at the time of their visit. In addition, confounders were identified by ICD codes and excluded when relevant, allowing for a stricter analysis of how age and diabetes may correlate with UTI visit characteristics. 

Limitations of our study include the use of a relatively small dataset of 167 visits (0.002% of the total estimated visits for the four years). Further dividing our dataset by age, diabetes status, and antibiotic prescriptions resulted in insufficient sample sizes within groups. As a result, we compared antibiotic prescription rates between all patients with T2DM versus non-T2DM without examining the effect of age and did not observe any difference. Furthermore, our study only included women with UTI codes and did not have a control group. Other limitations of our study were the lack of urine culture results, urinalysis results, and symptoms of UTI. This was the result of using the NAMCS database, which only provides visit data based on ICD codes and does not report bacterial strains from urine cultures, urinalysis results, or additional past medical history. Although this prevented our study from identifying bacterial and antibiotic resistance patterns of UTI visits across the nation, it did not weaken our analysis of UTI visits regarding age and T2DM status. However, further studies on UTI bacterial strains and antibiotic resistance are necessary. Despite these limitations of our study, our findings provide valuable insight into the demographics of UTIs by age group. Prevention, workup, and management of UTIs can be optimized by understanding which populations are especially vulnerable.

## Conclusions

This study conducted a national database analysis to investigate visits with a UTI diagnosis throughout the United States from 2014 to 2016 and 2018. The research aimed to identify patterns concerning age and Type 2 diabetes status in adult women. In the 167 surveyed visits, estimated to represent 7.4 million visits, there were no significant variations in T2DM rates and urinalysis rates between individuals aged <65 years and those aged ≥65 years. Additionally, there were no notable differences in urinalysis or antibiotic prescription rates between T2DM and non-T2DM patients. Conversely, patient race, ethnicity, region, and payer type all varied between the age groups. These findings suggest that the risk for UTIs is multifactorial and that providers are not increasing urinalysis rates and antibiotic prescription rates based on T2DM status before urine culture results. More research is needed to understand factors influencing UTI workup and management in women with T2DM. Such research could allow providers to better diagnose and treat UTIs in high-risk individuals, including older women and those with T2DM.
